# Predicting the molecular functions of regulatory genetic variants associated with cancer

**DOI:** 10.18632/oncotarget.28451

**Published:** 2023-08-30

**Authors:** Jun S. Song, Mohith Manjunath

**Keywords:** genome-wide association studies, cancer risk, regulatory variants, functional genomics

Some of inherited human genetic variation can contribute to important phenotypic diversity, such as the varying degrees of individual susceptibility to developing certain health conditions and individual response to therapeutic interventions. To date, over 490,000 genotype-phenotype associations have been discovered through large-scale genome-wide association studies (GWAS) [1]; however, molecular functions of most of these discovered GWAS variants remain unknown. There are several technical challenges hindering our understanding: (1) the effect size of a typical genetic variant, as measured in terms of the odds ratio of genotype occurrence in case versus control populations, is very small, suggesting that macroscopic systems-level phenotypic differences modulated by each variant may also be small and difficult to detect; (2) most reported variants reside in non-protein-coding regions of the human genome, indicating that they are likely affecting the regulation of some unknown target genes’ expression; and, (3) the discovered variants may not be functional themselves, but be merely in genetic linkage disequilibrium with other functional variants. A promising approach to address these challenges is to integrate genomic, epigenomic, transcriptomic and machine learning methods to identify functional genetic variants and characterize their mode of action in regulating target genes.

One particular mode of regulatory function amenable to this integrative analysis is altering the binding affinity of transcription factors (TF) to DNA recognition sequences [2]. That is, assuming that a causative variant perturbs the binding activity of a TF, one can focus on the variants that are genetically linked to a given GWAS variant and located in transcriptionally active open chromatin regions annotated via epigenomic profiling – e.g., DNase-seq, ATAC-seq, and histone modification signatures of enhancers and promoters, often available in public databases such as the Encyclopedia of DNA Elements (ENCODE), Roadmap Epigenomics Mapping Consortium (REMC) and Gene Expression Omnibus (GEO) [3–5]. The ability of these epigenomically filtered candidate variants to perturb the binding activity of a specific TF can then be assessed computationally by training machine learning algorithms on TF ChIP-seq and HT-SELEX-seq data to learn the salient features of preferred DNA recognition sequences and to predict how the variants in the context of surrounding nucleotides alter the strength of TF-DNA interaction [2, 6–12]. Allele-specific binding preferences of predicted TFs can be verified by searching for skewed allele frequencies of the candidate variants in raw ChIP-seq reads, appropriately taking into account potential mapping biases. Target genes that are differentially expressed between case and control populations as a result of the predicted perturbation of TF binding activity may then be identified via expression quantitative trait loci and allele-specific expression analyses using processed and raw RNA-seq data from The Cancer Genome Atlas (TCGA) and Genotype-Tissue Expression (GTEx) projects [13–15]; further support can be garnered by examining the allele-dependent correlation structure between target gene and TF mRNA levels and by utilizing chromatin conformation capture data providing evidence for looping between the candidate variant locus and predicted target gene promoter. This integrative approach can rapidly yield (functional variant, TF, target gene) triplets starting from cataloged GWAS variants [16, 17] and thus demonstrates that the microscopic effects of genetic variants on TF binding activity and target gene expression levels can be robustly predicted and measured, even though macroscopic phenotypic manifestations resulting from these microscopic alterations might get diluted by cellular network response and become difficult to detect.

**Figure 1:**
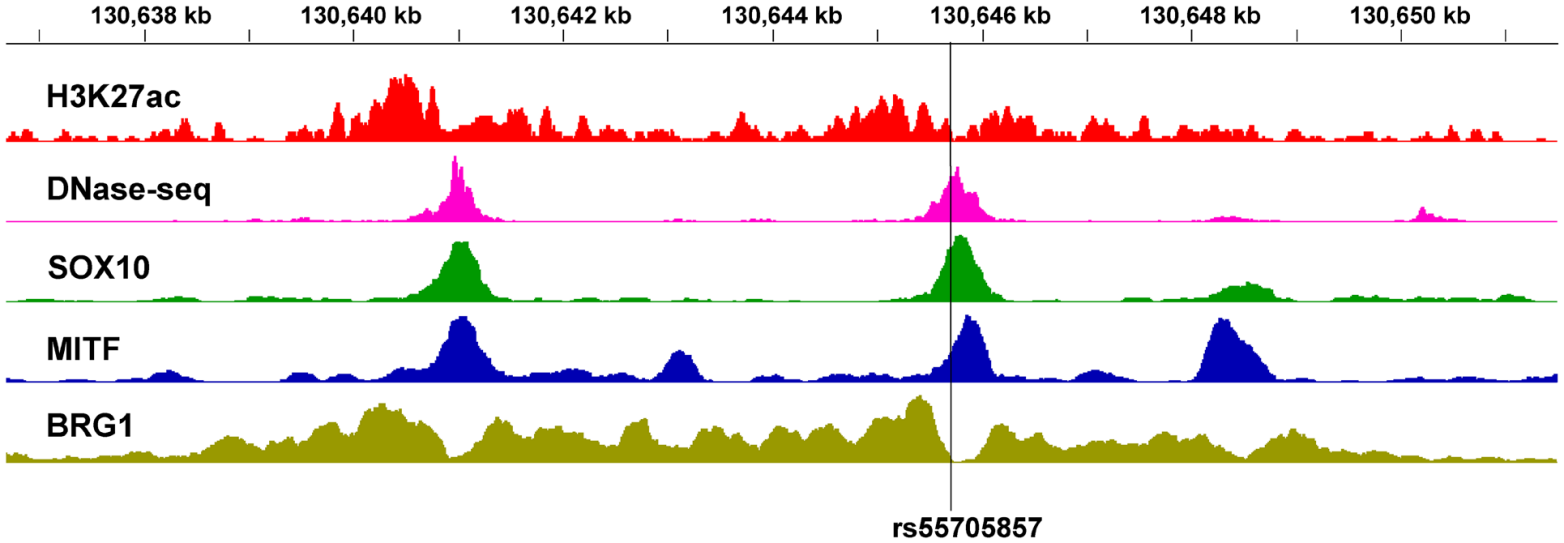
Chromatin landscape of the glioma GWAS locus rs55705857 in the melanocyte lineage. H3K27ac and DNase-seq data are in foreskin melanocyte primary cells (E059) [4]. SOX10, MITF and BRG1 ChIP-seq data are in the 501Mel melanoma cell line [22]. The chr8 coordinates are in hg19.

In some cases, GWAS variants may regulate a distal target gene that is very far away in genetic distance but brought to physical proximity via chromatin folding and looping. Critical examples relevant to cancer are found in the 8q24 locus, where several distal risk loci across multiple cancer types regulate the well-known oncogene *MYC* [18–20]. A recent striking example is the SNP rs55705857, which increases the risk of developing *IDH*-mutant low-grade glioma (LGG) by roughly 6-fold and modulates the transcription of *MYC*, located 1.9 Mb away [21]. It has been shown that the risk allele of rs55705857 directly disrupts the binding of OCT2/4 and also perturbs the nearby binding of SOX2 [21]. Intriguingly, the SNP is located in an evolutionarily conserved enhancer, the activity of which seems to be restricted to the brain and the melanocyte lineage. Our analysis of the publicly available ChIP-seq data in melanocytes [4] and the 501Mel melanoma cell line [22] shows that this same locus resides in a nucleosome-free region bound by MITF and SOX10, a paralog of SOX2 with important functions in neural crest-derived cells, and flanked by BRG1, a component of the PBAF chromatin remodeling complex (Figure 1). Furthermore, independent data from our previous study show that knocking down MITF in human primary melanocytes leads to reduction of H3K27ac at the enhancer and concomitant suppression of *MYC* expression [23], suggesting that this enhancer likely also regulates *MYC* transcription in the melanocyte lineage. Further investigation is needed to decipher whether the SNP rs55705857 similarly functions to modulate the risk of developing melanoma by altering *MYC* expression.

Large consortia, such as the ENCODE, REMC, TCGA and GTEx, have generated massive amounts of data greatly facilitating the functional characterization of human genetic variants. Effectively integrating these rich resources with GWAS results will continue to help prioritize causative inherited genetic variants and improve our molecular understanding of disease etiology, assisting the discovery of actionable genes to improve human health.
